# Identification of potential biological processes and key genes in diabetes-related stroke through weighted gene co-expression network analysis

**DOI:** 10.1186/s12920-023-01752-z

**Published:** 2024-01-02

**Authors:** Yong He, Yang Bai, Qin Huang, Jian Xia, Jie Feng

**Affiliations:** 1 Department of Neurology, Liuyang Jili Hospital, Changsha, Hunan China; 2 Department of Hematology and Critical Care Medicine, Central South University, The Third Xiangya Hospital, Changsha, China; 3 Department of Neurology, Peking University People’s Hospital, Beijing, 100044 China; 4 Department of Neurology, Xiangya Hospital, Central South University, Changsha, Hunan 410008 People’s Republic of China

**Keywords:** Stroke, Diabetes, Bioinformatics, Weight gene co-expression network analysis (WGCNA), Neutrophil extracellular trap formation (NETs)

## Abstract

**Background:**

Type 2 diabetes mellitus (T2DM) is an established risk factor for acute ischemic stroke (AIS). Although there are reports on the correlation of diabetes and stroke, data on its pathogenesis is limited. This study aimed to explore the underlying biological mechanisms and promising intervention targets of diabetes-related stroke.

**Methods:**

Diabetes-related datasets (GSE38642 and GSE44035) and stroke-related datasets (GSE16561 and GSE22255) were obtained from the Gene Expression omnibus (GEO) database. The key modules for stroke and diabetes were identified by weight gene co-expression network analysis (WGCNA). Gene Ontology (GO) and Kyoto Encyclopedia of Genes Genomes (KEGG) analyses were employed in the key module. Genes in stroke- and diabetes-related key modules were intersected to obtain common genes for T2DM-related stroke. In order to discover the key genes in T2DM-related stroke, the Cytoscape and protein–protein interaction (PPI) network were constructed. The key genes were functionally annotated in the Reactome database.

**Results:**

By intersecting the diabetes- and stroke-related crucial modules, 24 common genes for T2DM-related stroke were identified. Metascape showed that neutrophil extracellular trap formation was primarily enriched. The hub gene was granulin precursor (*GRN*), which had the highest connectivity among the common genes. In addition, functional enrichment analysis indicated that *GRN* was involved in neutrophil degranulation, thus regulating neutrophil extracellular trap formation.

**Conclusions:**

This study firstly revealed that neutrophil extracellular trap formation may represent the common biological processes of diabetes and stroke, and *GRN* may be potential intervention targets for T2DM-related stroke.

**Supplementary Information:**

The online version contains supplementary material available at 10.1186/s12920-023-01752-z.

## Introduction

Stroke is the second leading cause of death and disability worldwide, accounting for 17% of total deaths [1]. Type 2 diabetes (T2D) and ischemic stroke (IS) are common disorders that often arise together. Patients with diabetes have more than double risk of IS, relative to individuals without diabetes [2]. Despite significantly increased risk, there is a paucity of available treatments that specifically target the risk of stroke in subjects with diabetes [3]. Instead, current strategies for managing diabetes related stroke focus on the control of multiple risk factors, such as lipid profiles, blood pressure, smoking cessation, weight control, and glycemic management using lifestyle or drug interventions [3, 4]. Studying mechanism and downstream signaling of neuronal injury allows development of better stroke treatments. The effects of hyperglycaemia on the risk of cardiovascular disease are largely tissue-specific and pathway-specific. Impaired endothelial function, low-grade inflammation, AGEs, thrombosis, fibrinolysis and modifications of lipoprotein particles increase the risk of cardiovascular events [5]. The pathophysiology of diabetes-related stroke involves abnormalities in the endothelial, vascular smooth muscle cell, and platelet function [6]. Diabetes alters the structure and function of blood vessels, modulates immune function, and increases production of several prothrombotic factors. Over time, capillaries, arterioles, and arteries become increasingly stiff, tortuous, and narrowed [7]. The physiological processes critical to thrombogenesis, such as neuroinflammation, neuroplasticity, cerebral vasoreactivity, and blood–brain barrier (BBB) permeability, are compromised [8]. The molecular biological mechanisms by which diabetes exacerbates brain injury, however, has not been completely elucidated.

Detection of changes in gene expression about the process of diabetes and ischemic stroke using multiple functional genomic approaches can improve our understanding of the molecular mechanisms involved in T2DM-related stroke. But in the vast majority of cases, it focuses more on the effect of individual genes during analysis of gene differential expression, while ignoring the interaction of genes in complex biological gene networks, and fails to establish the relationship between illnesses and genes. Currently, the researches about WGCNA and diabetes were primarily focused on the function of pancreas [9], diabetic kidney [10], diabetic nephropathy [11], diabetic cardiomyopathy [12], and etc. Genetic factors occupy an irreplaceable role both in the pathogenesis of stroke and diabetes mellitus. Hence, exploring interactions at the gene level contributes to understand the correlation between diabetes and stroke. Weight gene co-expression network analysis (WGCNA) is an advanced analytical approach for discovering genetic network-disease relationship and gene–gene relationship, with the advantages of high sensitivity and system-level insight to genes with small fold change or low abundance [13]. The WGCNA approach has provided functional interpretation tools in system biology and has been increasingly used to construct co-expressed gene networks employed in the cardiovascular field [14, 15].

The purpose of this research was to reveal the biological processes of T2DM-related stroke by identifying the shared biological processes in stroke and diabetes co-expression networks. We identified key genes from the common genes of diabetes and stroke to pinpoint promising therapeutic targets for T2DM-related stroke.

## Materials and methods

### Dataset download

**Fig. 1:**
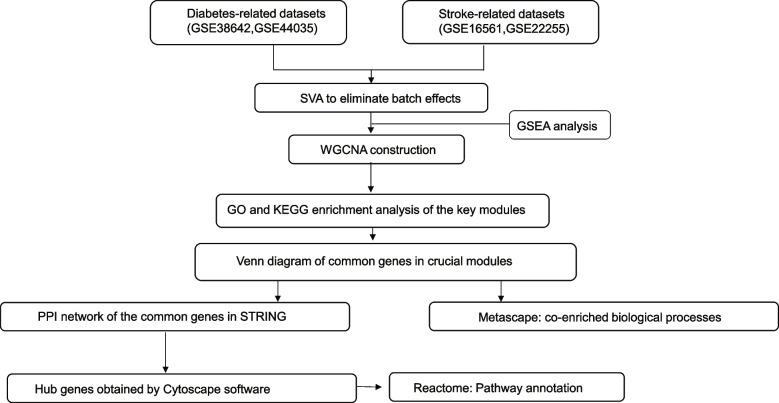
The research flowchart of data preparation and analysis. SVA: surrogate variable analysis; WGCNA: weighted gene co-expression network analysis; GSEA: Gene set enrichment analysis; GO: Gene Ontology; KEGG: Kyoto Encyclopedia of Genes and Genomes; PPI: protein–protein interaction

A flow chart for the present research is shown in Fig. 1. We used the term “ischemic stroke” and “diabetes” to search for ischemic stroke and diabetes’ gene expression profiles in GEO database (http://www.ncbi.nlm.nih.gov/geo/). The obtained mRNA microarray datasets were screened by the following criteria. First, these datasets must provide raw data that can be further analyzed. Second, profile information should include both case and control groups. Two stroke-related datasets (GSE38642 and GSE44035) and two diabetes-related datasets (GSE16561 and GSE22255) were chosen for next research (Table 1). The GSE38642 dataset comprised 54 non-diabetic controls and 9 diabetic patients and is based on the GPL6244 platform (Affymetrix Human Gene 1.0 ST Array). The GSE44035 dataset, which is also based on the GPL6244, comprised 9 non-diabetes controls and 1 patient with diabetes. In dataset GSE16561, which was produced on the GPL6883 platform (Illumina HumanRef-8 v3.0 expression beadchip), peripheral blood from 39 ischemic stroke patients and 24 healthy control subjects. Finally, the GSE22255 dataset was based on GPL570 platform (Affymetrix Human Genome U133 Plus 2.0 Array) and comprised 20 patients with ischemic stroke and 20 healthy controls.

**Table 1:** Data collection

Condition	Tissue	GEO dataset	Platform	Number of samples
Diabetes	Islets	GSE38642	GPL6244	63
Diabetes	Islets	GSE44035	GPL6244	10
Ischemic stroke	Peripheral blood	GSE16561	GPL6883	63
Ischemic stroke	Peripheral blood	GSE22255	GPL570	40

### Data preprocessing

Batch effects were removed with a time specific algorithm within LIMBR, based on Surrogate Variable Analysis (SVA) [16]. The results of batch effect elimination were presented through a PCA graph and box plot. Probe identifications (IDs) in the gene expression matrix were reannotated as gene symbols. Changes in gene expression levels were reported as log2 values. Subsequently, the stroke datasets were combined and a new gene expression profile for all samples was formed. The two stroke datasets were combined together into one new gene expression profile for all samples. Finally, we annotated the gene symbols of gene expression matrix as Entrez IDs by utilizing the org.Hs.eg.db package for subsequent analysis.

### Differential gene expression analysis

The limma package was applied to identify differentially expressed genes (DEGs) between case and control groups in diabetes and stroke with the following selection criteria: *P*-value of < 0.05, thresholds of |log FC|≥ 0.4. The hierarchical clustering analysis and volcano plot were represented by the R packages “pheatmap” and “ggplot2”, respectively.

### Gene set enrichment analysis

Gene set enrichment analysis (GSEA), based on functional categories, has been proved to be one of the most powerful and popular tools for analyzing gene enrichment pathway [17]. We used GSEA to compare the biological pathways between case and control groups. KEGG gene sets as Gene Symbols were chosen as the gene set database. The settings for the GSEA run include: (1) number of gene set permutations were set to 1000 and (2) collapse dataset to gene symbols = TRUE.

### Weight gene co-expression network analysis

Correlation networks are increasingly being used in bioinformatics applications. WGCNA is known as an algorithm in R-studio software for discovering the co-expressed gene modules, summarizing such clusters using an intramodular hub gene or the modules eigengene, relating modules to external sample traits, and calculating module membership measures. Correlated network based on gene screening methods can be used to identify candidate biomarkers [18]. In this research, we used WGCNA to create the co-expressed gene networks of diabetes and stroke. Firstly, we performed sample clustering to detect outliers. The “pickSoftThreshold” algorithm was used to select an appropriate soft threshold (β) and to obtain a biologically significant scale-free network (scale independence of > 0.8). Then gene–gene correlation matrix was built to describe the degree of association among nodes. The adjacency matrix was converted into a topological overlap matrix (TOM) using the “TOMsimilarity” algorithm. In order to identify co-expression modules, the gene hierarchical clustering dendrogram was obtained. The module eigengene (ME), as well as the correlation between clinical traits and ME were then calculated by spearman correlation analysis and hierarchical clustering to identify clinical-related modules.

### GO and KEGG enrichment analyses of genes in disease-related key modules

Gene ontology (GO) describes the overrepresented biological functions. Three independent ontologies accessible are being constructed: molecular function, cellular component, and biological process [19]. Kyoto Encyclopedia of Genes and Genomes (KEGG) enrichment analyses is a reference knowledge base for biological interpretation of large-scale molecular datasets, such as metagenome and genome sequences [20–23]. GO and KEGG analyses for diabetes and stroke related key modules were performed using the R clusterProfiler package.

### Detection of shared and key genes in T2DM-related stroke

The common genes of T2DM-related stroke were discovered by intersecting diabetes-related crucial modules with stroke-related crucial modules. Furthermore, the STRING website (https://string-db.org/) was used to build the protein–protein interaction (PPI) relationships. STRING (version 11.5) covers 67,592,464 proteins from 14094 organisms and 20,052,394,042 interactions. We selected the gene symbol as input of website https://cn.string-db.org/, then chose multiple proteins, used gene symbol as list of names, and select homo sapiens as organism. The cytoHubba plug-in in Cytoscape (version 3.9.1) was employed to obtain the hub genes of T2DM-related stroke.

### Enriched biological processes of common and crucial genes in T2DM-related stroke

The GO vocabularies, which include biological processes (BPs), molecular functions (MFs), and cellular components (CCs), were performed using Metascape (http://metascape.Org/gp/) to find significantly enriched terms (*P* value ≤ 0.01) [24]. The biological pathways associated with hub genes were annotated and visualized using Reactome Database (https://reactome.org), which is an open-access, open-source, peer-reviewed, and manually curated database [25]. The R code is available in Supplementary material.

## Results

### Integrated screening for genes and GSEA analysis in diabetes and stroke

**Fig. 2:**
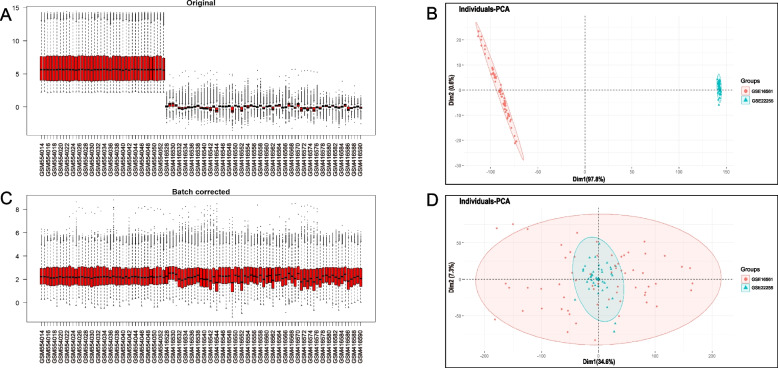
Batch effects considered in analysis. **A**, **B** the distribution of stroke samples before elimination of batch effect. **C**, **D** the distribution of stroke samples after eliminating the batch effect

**Fig. 3:**
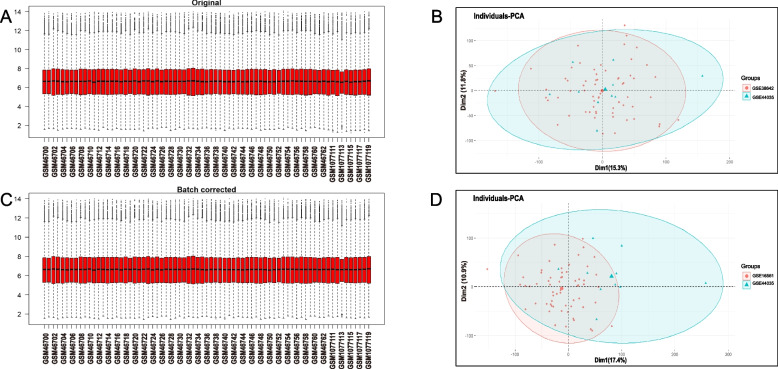
Batch effects considered in analysis. **A**, **B** the distribution of diabetes samples before elimination of batch effect. **C**, **D** the distribution of diabetes samples after eliminating the batch effect

**Fig. 4:**
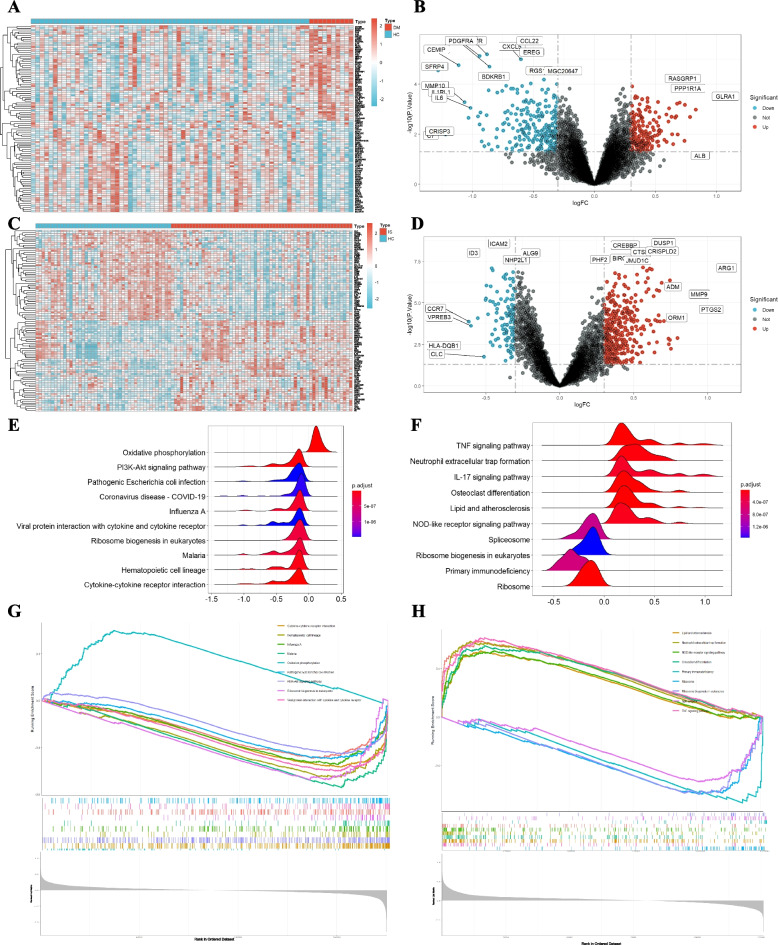
Identification and pathway analyses of differentially expressed genes (DEGs). **A** Heatmap of DEGs in diabetes related datasets; **B** Volcano plots showing the differential genes in diabetes related dataset. **C** Heatmap of DEGs in stroke related datasets; **D** Volcano plots showing the differential genes in stroke related dataset. **E**, **F** Ridgeline plot showing KEGG pathways enrichment in diabetes (**E**) and stroke (**F**). **G**, **H** Gene set enrichment analysis (GSEA) plots showing the most enriched gene sets of all detected genes in the diabetes (**G**) and stroke (**H**) samples

Four GEO datasets were used for the identification of diabetes and stroke-associated genes (Table 1). As shown in Figs. 2 and 3, batch effects have been removed by sva package in all samples from the stroke and diabetes datasets. We then used “limma” R package to identified differentially expressed genes (DEGs) between case and control group in diabetes and stroke. In total, 81 upregulated genes and 153 downregulated genes were included in patients with diabetes (Fig. 4A and B). 158 upregulated genes and 29 downregulated genes were included in IS patients (Fig. 4C and D) based on the criteria *p* < 0.05 and |logFC|≥ 0.4. GSEA was used to reveal the potential molecular mechanisms of diabetes and stroke based on all gene information in the gene expression matrix. The most highly enriched pathways by enrichment score in diabetes related datasets were related to oxidative phosphorylation, ribosome biogenesis in eukaryotes, hematopoietic cell lineage, and cytokine-cytokine receptor interaction (Fig. 4E and G). The enrichment analysis of gene sets about stroke revealed that compared to control samples, TNF signaling pathway, neutrophil extracellular trap formation, IL-17 signaling pathway, lipid and atherosclerosis, ribosome biogenesis in eukaryotes, and primary immunodeficiency (Fig. 4F and H). According to GSEA analysis, ribosome biogenesis in eukaryotes was the common biological pathway relevant to the pathogenesis of diabetes and stroke.

### Stroke-related key module identification

**Fig. 5:**
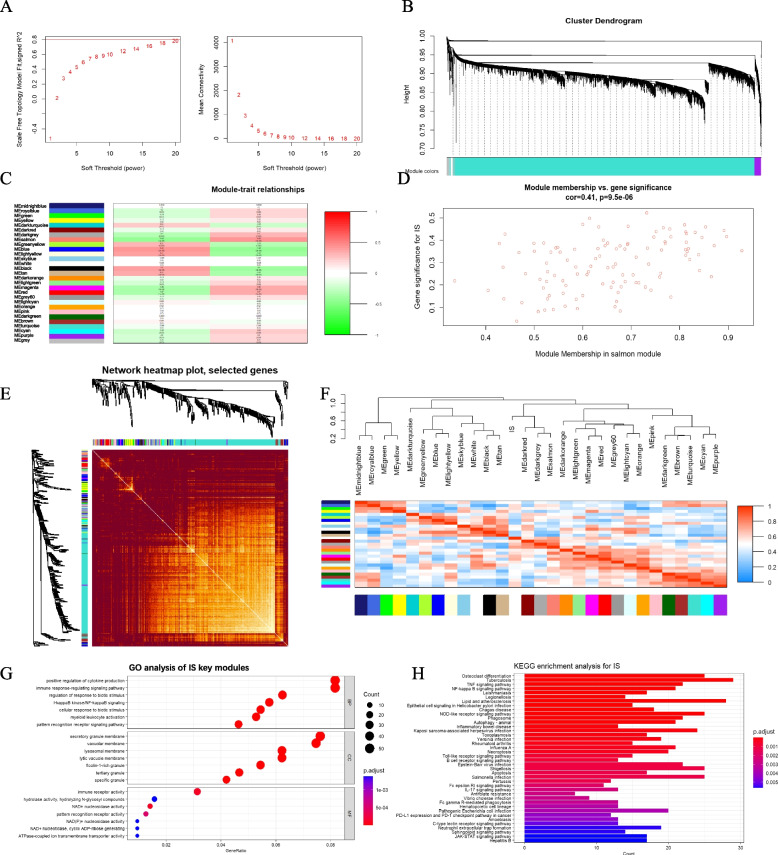
Construction of weighted co-expression network for stroke-related datasets. **A** Network topology analysis of different soft threshold power. **B** Dendrograms of genes acquired by mean linkage hierarchical clustering. **C** The relationship between trait and modules. Correlation coefficients and corresponding *P* value are listed for each module. **D** The genes in the salmon module were significantly correlated with stroke. **E** Heatmap depicts the Topological Overlap Matrix (TOM) of genes selected for weighted co-expression network analysis. **F** Heatmaps and hierarchical cluster dendrograms of clinical traits and module eigengene. **G**, **H** The GO and KEGG pathways in key modules

In stroke datasets, the appropriate soft-thresholding power was chosen based on the number of samples to make the resulting networks conservative (if number of samples ≥ 40, unsigned and signed hybrid networks = 6). Network topology analysis of different soft threshold power was shown in Fig. 5A. Each module was represented by a different color. Based on spearman coefficient, the relationships between modules were assessed through a heatmap about module-trait relationships (Fig. 5B and C). The heatmaps of the correlation between values of clinical features and module eigengene showed that the salmon (*r* = 0.44, *p* < 0.05), darkgrey (*r* = 0.3, *p* = 0.002), red (*r* = 0.37, *p* < 0.05), and magenta (*r* = 0.36, *p* < 0.05) modules were highly positively related with stroke (Fig. 5D-F). As a result, the four modules were classified as stroke-related crucial modules. GO and KEGG analyses were performed on the genes of key modules (Fig. 5G and H). The GO analyses demonstrated that these genes were primarily related to positive regulation of cytokine production, immune response-regulating signaling pathway, regulation of response to biotic stimulus, NF-kappa B signaling, cellular response to biotic stimulus, and myeloid leukocyte activation in biological processes. In terms of cell components, genes were primarily enriched in secretory granule membrane, vacuolar membrane, lysosomal membrane, lytic vacuole membrane, and ficolin-1-rich granule. Furthermore, molecular function was primarily enriched in the immune receptor activity, hydrolase activity, NAD + nucleosidase activity, pattern recognition receptor activity, and ATPase-coupled ion transmembrane transporter activity. Next, we performed KEGG analysis on the genes in the four modules (Fig. 5H). The findings revealed an association between the TNF signaling pathway, NF-kappa B signaling pathway, lipid and atherosclerosis, toll-like receptor signaling pathway, apoptosis, neutrophil extracellular trap formation.

### Diabetes-related key module identification

**Fig. 6:**
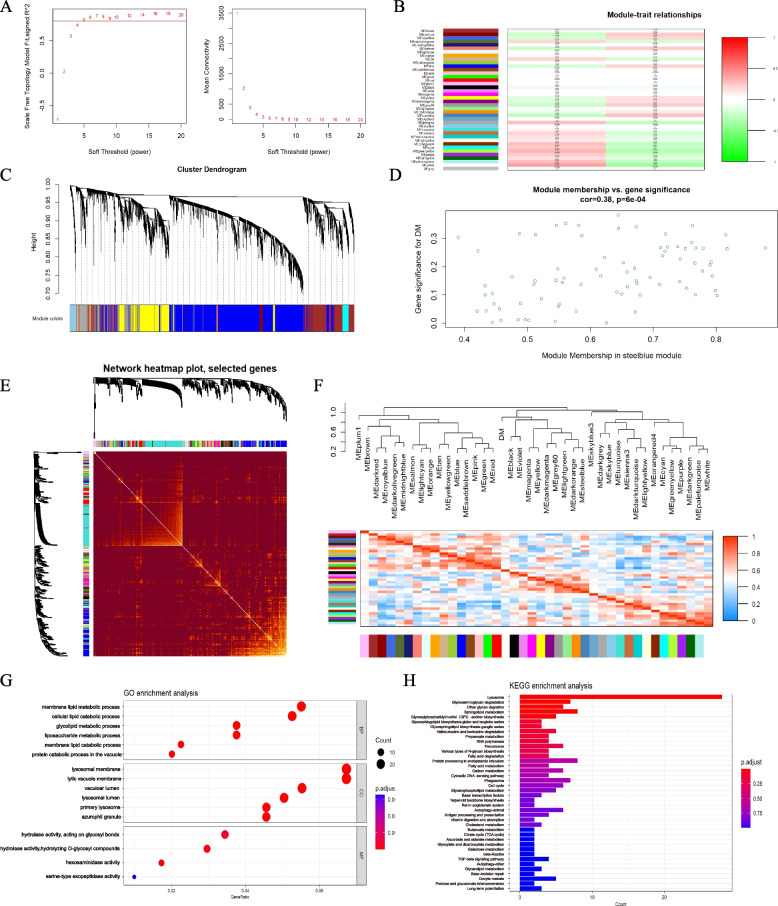
Construction of weighted co-expression network for diabetes-related datasets. **A** The value of scale independence on the left and the value of mean connectivity on the right. **B** The relationship between trait and modules. Correlation coefficients and corresponding P value are listed for each module. **C** The cluster dendrogram of all gene were grouped into different modules. **D** The genes in the steelblue module were significantly correlated with diabetes. **E** Network heatmap of all genes (a color change from red to yellow indicates a high degree of overlap between modules). **F** Heatmaps and hierarchical cluster dendrograms of clinical traits and module eigengene. **G**, **H** The GO and KEGG pathways in key modules

In the stroke and diabetes-related datasets, we found co-expressed gene modules using WGCNA. As illustrated in Fig. 6A, the scale-free topological index was 0.8 when the soft thresholds for diabetes was 6. The derived gene dendrograms and corresponding module colors were showed in Fig. 6B and C. The correlations between the two phenotypes (disease and health states) were calculated by the hierarchical clustering and spearman correlation analyses. Three modules “steelblue”, “dark red”, and “Grey60” had a highly positive association with diabetes, which were chosen as diabetes-related modules (steelblue: *r* = 0.28, *p* = 0.02; dark red: *r* = 0.23, *p* = 0.05; Grey60: *r* = 0.24, *p* = 0.05) (Fig. 6D-F). We conducted GO and KEGG enrichment analysis on the genes included diabetes-related key modules, as well as pathway annotation of key diabetes-related genes, to discover the underlying molecular biological process of diabetes (Fig. 6G and H). The GO terms of biological processes showed that they were primarily enriched in membrane lipid metabolic process, cellular lipid catabolic process, glycolipid metabolic process, and liposaccharide metabolic process (Fig. 6G). With regard to cellular components, these genes were primarily enriched in lysosomal membrane, lytic vacuole membrane, vacuolar lumen, lysosomal lumen, and primary lysosome. In terms of molecular function, these genes were primarily enriched in hydrolase activity, hexosaminidase activity, and serine-type exopeptidase activity. KEGG enrichment analysis suggested that they were mostly involved in pathways of lysosome, glycosaminoglycan degradation, sphingolipid metabolism, phagosome, and autophagy (Fig. 6H).

### The shared genes and functional enrichment analysis in diabetes and stroke

**Fig. 7:**
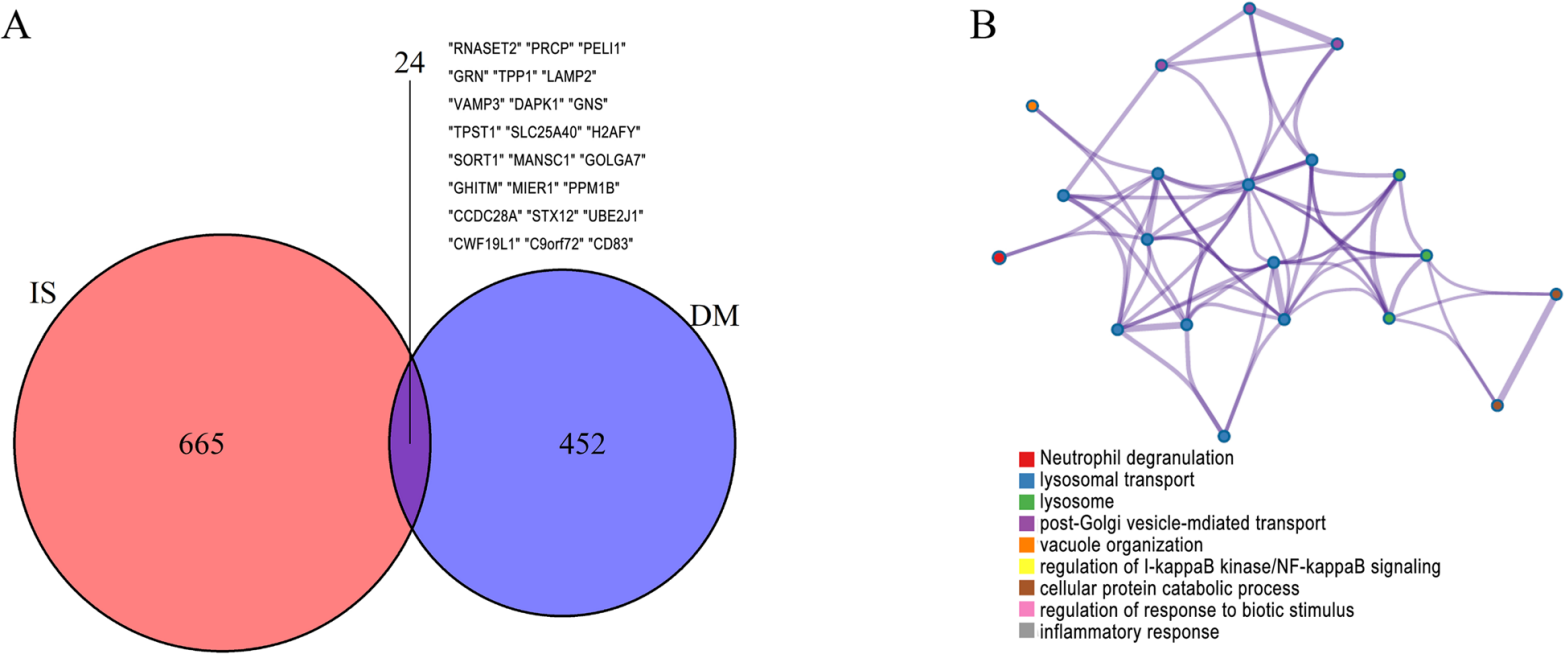
Screening of the common genes between stroke and diabetes. **A** The common genes screened by intersecting the diabetes and stroke related modules. **B** The co-enriched biological processes by metascape

A total of 689 and 476 genes were identified from stroke and diabetes-related modules, respectively. 24 genes were selected by taking the intersection of stroke and diabetes-related genes (Fig. 7A), which were considered to be extremely associated with the pathogenesis T2DM-related stroke. To investigate potential roles of the shared genes in diabetes and stroke, we used metascape to analyze the enriched pathway. As shown in Fig. 7B, the results revealed that these genes were enriched in various biological activities including neutrophil degranulation, lysosomal transport, regulation of NF-kappa B signaling, and inflammatory response. The most enriched ontology clusters belonged to neutrophil degranulation (logP = -53.25, log(q-value) = -48.904).

### Identification of key genes in DM-related stroke

**Fig. 8:**
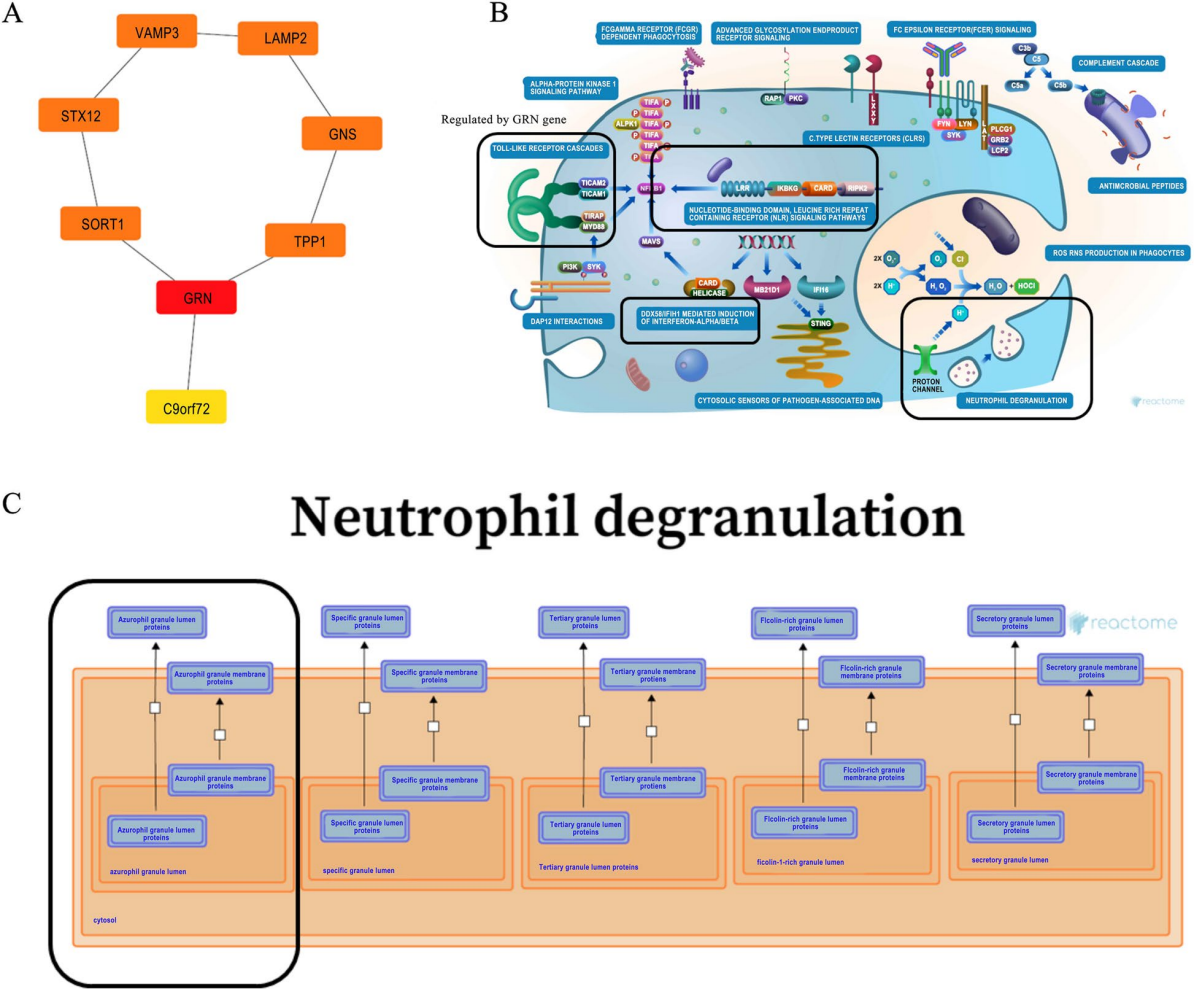
Screening the hub genes and the related biological process analysis. **A** a PPI network of common genes constructed by Cytoscape software. **B**, **C** the role of hub gene GRN regulatory pathways in inflammatory response (**B**) and neutrophil degranulation (**C**). The schematic art pieces were provided by the Reactome Database (www.reactome.org) under Creative Commons Attribution 4.0 License

In terms of the 24 shared genes obtained by a disease module-module intersection, we used the STRING database to construct PPI networks (Fig. 8A). Next, cytoHubba in Cytoscape software was used to screen for and visualize the hub genes in the network. As shown in Fig. 8A, *GRN* (granulin precursor), was identified as the hub gene in T2DM-related stroke. To further understanding how *GRN* acts in DM-related stroke, the website Reactome was used to analyze its regulatory pathways. We used human reactome database and found that *GRN* was involved in Toll-like Receptor Cascades, Neutrophil degranulation, DDX58/IFIH1-mediated induction of interferon-alpha/beta, and NLR signaling pathway (Fig. 8B). Notably, GRN was involved in exocytosis of azurophil granule lumen proteins, which contributed to neutrophil degranulation (Fig. 8C). Thus, we believe that *GRN* might participate in inflammatory response and neutrophil extracellular trap formation by regulating exocytosis of azurophil granule lumen proteins in T2DM-related stroke.

## Discussion

We used the WGCNA package to investigate key genes and probable biological processes associated with DM-related stroke in this study. Twenty- four common genes were identified to be present in both stroke and diabetes-related crucial modules, indicating that they are the most likely to participate in biological function in T2DM-related stroke. The common genes were enriched highly in biological processes of neutrophil degranulation, regulation of response to biotic stimulus, and inflammatory response, which was associated with the results of GSEA analysis in stroke-related datasets. Most importantly, among these common genes, *GRN* was identified as the hub gene of DM-related stroke. Functional annotation revealed that *GRN* acted in T2DM-related stroke by regulating neutrophil degranulation.

Variations in gene expression signatures provide novel insight to the mechanism of DM-related stroke and assists in finding intervention targets. Sentinel variants at *PEAR1* and *RGS18* were associated with thrombosis risk through a whole genome sequencing, which provide insights regarding the mechanism by genetics may influence cardiovascular disease risk [26]. The mRNA expression of platelet activating factor receptor (PAFR) was significantly higher in patients with diabetes, which might cause macrovascular disease through impaired endothelial function [27]. The study analyzing high-throughput gene expression in blood samples has shown that the circulating gene expression makers including *CREM*, *PELI1*, and *ZAK* were verified to be up-regulated in cardioembolic stroke [28]. In current study, we performed gene co-expression pattern analysis (WGCNA), rather than individual gene/protein-focused analytic strategy, for evaluating expression similarity among genes of T2DM-related stroke. Many of these common genes were involved in immune effector process, apoptosis, and necroptosis. Death-associated protein kinase 1 (*DAPK1*), a Ca2 + /calmodulin (CaM)-dependent serine/threonine protein kinase, plays important roles in diverse apoptosis pathways in neuronal cell death [29]. A quantitative proteomic analysis revealed *DAPK1* as the most prevalent protein recruited to the cytoplasmic tail of glutamate receptor during cerebral ischemia [30]. Targeting *DAPK1*-related pro-death signals could be considered as a promising therapeutic approach in salvageable brain tissue after ischemic stroke. Sortilin 1 (*SORT1*) was one of CVD-risk loci identified with genome-wide association studies (GWAS) over the last decade [31]. Sortilin, encoded by *SORT1*, serving as a key receptor for lipids, cytokines, and enzymes and participating in pathological cargo loading to and trafficking of extracellular vesicles, is also known for its functional role in metabolic disorders and cardiovascular disease [32]. The multiple contributions of sortilin to cardiovascular risk suggest it as a potential drug target. in addition, other common genes identified in our study such as *PELI1*, *LAMP2*, and *PPM1B*, were linked to cell death and inflammation [33–35]. Thus, preventing aberrant cell death and maintaining cellular homeostasis is important to aid in the development of drugs for T2DM-related stroke. Furthermore, we measured correlation between diseases and highly co-expressed gene sets. Compared to conventional differential expression analysis, it is a more effective technique for detecting gene expression with low variation and abundance, as well as being less prone to information loss [36]. Quite a few researches have demonstrated the value of WGCNA in systematically identifying critical genes and relevant mechanisms about stroke or diabetes [36–39].

In our study, we discovered key biological processes in the common genes of diabetes and stroke, including neutrophil degranulation, regulation of response to biotic stimulus, and inflammatory response. Consistently, neutrophil extracellular trap formation (NETs) was highly enriched in both GSEA analysis and function enrichment analysis of stroke-related key modules. This finding indicated that neutrophil extracellular trap formation might be a crucial mechanism for DM-related stroke, explaining the role of neutrophils in pathogenesis for stroke patients with diabetes. Neutrophils, the first line of defense under inflammation and infection, can release their decondensed chromatin and form large extracellular DNA networks [40]. NETs formation mediates the propagation of thrombosis and inflammation and, thereby contributes to stroke. Several studies have demonstrated that NETs contributed to the composition of ischemic stroke thrombi [41, 42] and surrogate markers of NETs in plasma including circulating citrullinated histone H3 (H3Cit) and cell-free DNA (cfDNA) were associated with ischemic stroke outcomes [43, 44]. Hyperglycemia is associated with poor outcome in acute ischemic stroke patients. Recent studies have shown that neutrophil extracellular trap formation in serum of diabetes patients can activate vascular endothelial cells and thrombosis. Cell death and neutrophil activation are considered to be key factors producing endothelial injury under the condition of diabetes, and NETs formation further accelerated the vascular injure [45]. Blocking NET formation has significantly reduced brain infarctions and improved outcomes in diabetic mice [46]. A prominent characteristic of ischemic stroke is platelet activation and immunothrombosis stimulated by NETs [47]. Therefore, NETs formation promotes hypercoagulability and induces ischemic stroke in patients with diabetes, which is a potential biological process of T2DM-related stroke.

*GRN* (granulin), as a protein coding gene, was expressed in immune cells and epithelia and regulated biological functions including cell proliferation, inflammation, and wound healing [48]. Interactions between inflammation and *GRN* are complex. Progranulin (PGRN) can bind receptors of TNF-α and block the role of TNF-α to stimulate neutrophile respiratory burst. Therefore, *PGRN* is considered as anti-inflammatory factor [49]. While this anti-inflammatory activity was degraded by NE-mediated proteolysis of *PGNR* to *GRN* peptides [50]. *GRN* was involved in a pro-inflammatory response at the early stages after cerebral ischemia. Neutrophiles are infiltrated in brain parenchyma after ischemia stroke and the increased activity of neutrophil elastase cleaves *PGRN* into *GRN* peptide, exacerbating the inflammation and tissue damage after ischemia [51]. *GRN* has also been detected in neutrophil-rich peritoneal exudates, induce the release of neutrophil-attracting IL-8 from epithelial cells, and may enhance inflammation [52]. It can also modulate inflammation in neurons by preserving neuronal integrity, axonal outgrowth, and neurons survival [53]. In acute injury and inflammation, *GRN* is important in the initiation of inflammation by recruiting neutrophils, macrophages, and fibroblasts [54]. *GRN*, at elevated levels, has been associated with poor prognosis in infectious diseases. Serum *GRN* levels in sepsis exhibited positively correlation with inflammatory factors including hypersensitive C-reactive protein and procalcitonin [55]. The cell counts of neutrophils and macrophages were increased in transcutaneous puncture wounds under administration of *GRN* [56]. Elevated *PGRN* levels may reflect increased pro-inflammatory *GRN* levels because *PGRN* and *GRN* are indistinguishable from each other in ELISA [57]. In addition, several studies have shown that *PGRN* has proinflammatory effects in diabetes and obesity. *PGRN* played a role as a novel marker for chronic inflammation associated with diabetes in human [58]. Consistently, *PGRN* levels were increased in white adipose tissue of the mice fed with a high-fat-diet (HFD) and exerted pro-inflammatory functions [59]. Therefore, *PGRN* may have two absonant effects, depending on the tissue microenvironment and disease stage. In our study, *GRN* was shown to be the hub gene for key diabetes and stroke-related modules, suggesting that *GRN* plays critical roles in DM-related stroke. According to functional annotation, the participation of *GRN* in the regulation of neutrophil extracellular trap formation and inflammatory response, *GRN* plays a role in T2DM-related stroke.

## Conclusions

A total of 24 common genes may be involved in the mechanisms of T2DM-related stroke through their involvement in neutrophil degranulation, lysosomal transport, regulation of NF-kappa B signaling, and inflammatory response. More crucially, *GRN* participating in NETs formation may be promising intervention targets for T2DM-related stroke. However, the current research had several limitations. First, the sample size of patients with stroke and diabetes in the GEO database is relatively small. Although we have eliminated batch effects to integrate information from different datasets, studies with larger sample sizes are still needed in subsequent analysis and validation. Second, considering that stroke is a serious complication with type 2 diabetes, our study primarily investigates the molecular mechanism network between type 2 diabetes and stroke, while there is insufficient evidence to support the molecular mechanism network between type 1 diabetes and stroke. Third, the findings in this study are based on bioinformatics analysis. The relationship between GRN and DM-related stroke still needs to be verified by more in vitro and in vivo experiments. Further basic research and clinical research should be focused on a better understanding of the pathophysiological mechanism of GRN in T2DM-related stroke and may contribute to DM-related stroke therapy.

### Supplementary Information

**Additional file 1:** **R code 1.** Integrated screening for genes and GSEA analysis in diabetes and stroke. **R code 2.** Stroke-related key module identification. **R code 3.** Diabetes-related key module identification.

## Data Availability

All data from the GSE38642, GSE44035, GSE16561, and GSE22255 datasets are available at the GEO database (https://www.ncbi.nlm.nih.gov/geo/).
