# Supplementary material for: Identification of potential biological processes and key genes in diabetes-related stroke through weighted gene co-expression network analysis
Source: BMC Med Genomics. 2024 Jan 2;17:8. doi: 10.1186/s12920-023-01752-z (PMC10762844; doi:10.1186/s12920-023-01752-z)
Supplement: Supplementary file 1 — Additional file 1: R code 1. Integrated screening for genes and GSEA analysis in diabetes and stroke. R code 2. Stroke-related key module identification. R code 3. Diabetes-related key module identification. [file 12920_2023_1752_MOESM1_ESM.docx]

**Supplemental online content**

**R code 1: Integrated screening for genes and GSEA analysis in diabetes and stroke.**

**R code 2: Stroke-related key module identification.**

**R code 3: Diabetes-related key module identification.**

**R code 1: Integrated screening for genes and GSEA analysis in diabetes and stroke**

rm(list=ls())

library(GEOquery)

getwd()

setwd("F:/r")

eSet <- getGEO("GSE22255",

destdir = '.',

getGPL = F)

exp <- exprs(eSet[[1]])

GPL=getGEO(filename = 'GSE22255_family.soft.gz')

gpl=GPL@gpls[[1]]@dataTable@table

colnames(gpl)

ids=gpl[,c(1,11)]

write.csv(ids,file = "ids.csv")

colnames(ids) = c("probe_id" ,"symbol")

exp=as.data.frame(exp)

exp$probe_id=rownames(exp)

exp3=merge(exp,ids,by.x="probe_id", by.y="probe_id")

exp3=exp3[!duplicated(exp3$symbol),]

rownames(exp3)=exp3$symbol

exp3=exp3[,-c(1)]

save(exp3,file = 'Expreset22255.Rdata')

load("Expreset22255.Rdata")

load("Expreset16561.Rdata")

library(dplyr)

merge_eset<-inner_join(exp3,exp2,by="symbol")

rownames(merge_eset)=merge_eset$symbol

merge_eset<-merge_eset[,-c(41)]

exp=as.matrix(merge_eset)

data1<-read.table("ids.csv",sep=',',header=T)

modcombat = model.matrix(~1, data = data1)

batch = data1$batch

combat_edata = ComBat(dat=merge_eset, batch=batch, mod=modcombat,par.prior=TRUE, prior.plots=FALSE)

write.table(combat_edata, "ComBat_data(all)(counts).txt", sep = "\t", quote = F)

n <- as.data.frame(t(merge_eset))

name <- row.names(n)

par(las=2,cex.lab=0.5,col="black")

boxplot(as.data.frame(merge_eset),cex=0.2,las=2,col="red",main="Original",labels = name)

boxplot(as.data.frame(combat_edata),cex=0.2,las=2,col="red",main="Batch corrected",labels=name)

library(sva)

library(bladderbatch)

pca_plot = function(dddd,ggggg){

library("FactoMineR")

library("factoextra")

df.pca <- PCA(t(dddd), graph = FALSE)

fviz_pca_ind(df.pca,

#axes = c(2,3),

geom.ind = "point",

col.ind = ggggg ,

addEllipses = TRUE,

legend.title="Groups"

)

}

data1<-read.table("ids.csv",sep=',',header=T)

pca_plot(combat_edata,factor(data1$GSE))

pca_plot(merge_eset,factor(data1$GSE))

rm(list=ls())

library(GEOquery)

getwd()

setwd("F:/r")

eSet <- getGEO("GSE25724",

destdir = '.',

getGPL = F)

exp <- exprs(eSet[[1]])

exp=as.data.frame(exp)

exp$probe_id=rownames(exp)

library(BiocManager)

if (!requireNamespace("hgu133a.db", quietly = TRUE))

BiocManager::install("hgu133a.db")

library(hgu133a.db)

ids1=toTable(hgu133aSYMBOL)

write.csv(ids1,file = "ids1.csv")

colnames(ids1) = c("probe_id" ,"symbol")

exp6=merge(exp,ids1,by.x="probe_id", by.y="probe_id")

exp6=exp6[!duplicated(exp6$symbol),]

rownames(exp6)=exp6$symbol

exp6=exp6[,-c(1)]

save(exp6,file = 'Expreset25724.Rdata')

getwd()

setwd("F:/r")

load("Expreset25724.Rdata")

load("Expreset44035.Rdata")

load("Expreset38642.Rdata")

library(dplyr)

merge_eset<-inner_join(exp5,exp4,by="symbol")

rownames(merge_eset)=merge_eset$symbol

merge_eset<-merge_eset[,-c(64)]

exp=as.matrix(merge_eset)

data1<-read.table("batch.csv",sep=',',header=T)

modcombat = model.matrix(~1, data = data1)

batch = data1$batch

library(sva)

library(bladderbatch)

combat_edata = ComBat(dat=merge_eset, batch=batch, mod=modcombat,par.prior=TRUE, prior.plots=FALSE)

write.table(combat_edata, "ComBat_data(all)(counts)DM.txt", sep = "\t", quote = F)

n <- as.data.frame(t(merge_eset))

name <- row.names(n)

par(las=2,cex.lab=0.5,col="black")

par(mar=c(6,2,1,1))

boxplot(as.data.frame(merge_eset),cex=0.2,las=2,col="red",main="Original",labels = name)

boxplot(as.data.frame(combat_edata),cex=0.2,las=2,col="red",main="Batch corrected",labels=name)

pca_plot = function(dddd,ggggg){

library("FactoMineR")

library("factoextra")

df.pca <- PCA(t(dddd), graph = FALSE)

fviz_pca_ind(df.pca,

#axes = c(2,3),

geom.ind = "point",

col.ind = ggggg ,

addEllipses = TRUE,

legend.title="Groups"

)

}

data1<-read.table("batch.csv",sep=',',header=T)

pca_plot(combat_edata,factor(data1$GSE))

pca_plot(merge_eset,factor(data1$GSE))

rm(list = ls())

options(stringsAsFactors = F)

getwd()

setwd("F:/r")

library(ggplot2)

library(limma)

library(pheatmap)

library(ggsci)

library(dplyr)

rt<-read.table("comBatstroke.csv",sep=',',header=T)

data=rt

row.names(data)<-data$X

data<-data[,-c(1)]

HC=44

HCData=data[,as.vector(colnames(data)[1:HC])]

IS=HC+1

ISData=data[,as.vector(colnames(data)[IS:ncol(data)])]

rt=cbind(HCData,ISData)

HCNum=ncol(HCData)

ISNum=ncol(ISData)

Type=c(rep("HC",HCNum),rep("IS",ISNum))

design <- model.matrix(~0+factor(Type))

colnames(design) <- c("HC","IS")

fit <- lmFit(rt,design)

cont.matrix<-makeContrasts(IS-HC,levels=design)

fit2 <- contrasts.fit(fit, cont.matrix)

fit2 <- eBayes(fit2)

Diff=topTable(fit2,adjust='fdr',number=length(rownames(data)))

DIFFOUT=rbind(id=colnames(Diff),Diff)

write.table(DIFFOUT,file="DIFF_all.IS.xls",sep="\t",quote=F,col.names=F)

Diff=Diff[order(as.numeric(as.vector(Diff$logFC))),]

diffGene=as.vector(rownames(Diff))

diffLength=length(diffGene)

afGene=c()

if(diffLength>(100)){

afGene=diffGene[c(1:50,(diffLength-50+1):diffLength)]

}else{

afGene=diffGene

}

afExp=rt[afGene,]

n=t(scale(t(afExp)))

n[n>2]=2

n[n< -2]= -2

Type=c(rep("HC",HCNum),rep("IS",ISNum))

names(Type)=colnames(rt)

Type=as.data.frame(Type)

anncolor=list(Type=c(IS=pal_npg()(1),HC=pal_npg()(2)[2]))

##pdf(file="DIFFdm_heatmap.pdf",height=7,width=8)

pheatmap(n,

annotation=Type,

color = colorRampPalette(c(pal_npg()(2)[2],"white", pal_npg()(1)))(50),

cluster_cols =F,

show_colnames = F,

scale="row",

fontsize = 8,

fontsize_row=3,

fontsize_col=8,

annotation_colors=anncolor

)

#dev.off()

adjP=0.05

aflogFC=0.5

Significant=ifelse((Diff$P.Value<adjP & abs(Diff$logFC)>aflogFC), ifelse(Diff$logFC>aflogFC,"Up","Down"), "Not")

p = ggplot(Diff, aes(logFC, -log10(P.Value)))+

geom_point(aes(col=Significant),size=3)+

scale_color_manual(values=c(pal_npg()(2)[2], "#838B8B", pal_npg()(1)))+

labs(title = " ")+

theme(plot.title = element_text(size = 16, hjust = 0.5, face = "bold"))+

geom_hline(aes(yintercept=-log10(P)), colour="gray", linetype="twodash",size=1)+

geom_vline(aes(xintercept=aflogFC), colour="gray", linetype="twodash",size=1)+

geom_vline(aes(xintercept=-aflogFC), colour="gray", linetype="twodash",size=1)

Diff$symbol=rownames(Diff)

#pdf("DIFF_vol.pdf",width=5.5,height=5)

p=p+theme_bw()

p+geom_point(size = 2, shape = 1,) +

ggrepel::geom_label_repel(

aes(label = symbol),

color="black",

label.size =0.1

)

#dev.off()

rm(list = ls())

options(stringsAsFactors = F)

getwd()

setwd("F:/r")

library(ggplot2)

library(limma)

library(pheatmap)

library(ggsci)

library(dplyr)

rt=read.table("ComBat_data(all)(counts)DM.txt",sep="\t",header=T,check.names=F)

write.table(rt,"comBatDM.csv",row.names=TRUE,col.names=TRUE,sep=",")

rt<-read.table("comBatDM.csv",sep=',',header=T)

data=rt

row.names(data)<-data$X

data<-data[,-c(1)]

HC=63

HCData=data[,as.vector(colnames(data)[1:HC])]

DM=HC+1

DMData=data[,as.vector(colnames(data)[DM:ncol(data)])]

rt=cbind(HCData,DMData)

HCNum=ncol(HCData)

DMNum=ncol(DMData)

Type=c(rep("HC",HCNum),rep("DM",DMNum))

design <- model.matrix(~0+factor(Type))

colnames(design) <- c("HC","DM")

fit <- lmFit(rt,design)

cont.matrix<-makeContrasts(DM-HC,levels=design)

fit2 <- contrasts.fit(fit, cont.matrix)

fit2 <- eBayes(fit2)

Diff=topTable(fit2,adjust='fdr',number=length(rownames(data)))

DIFFOUT=rbind(id=colnames(Diff),Diff)

write.table(DIFFOUT,file="DIFF_all.DM.xls",sep="\t",quote=F,col.names=F)

Diff=Diff[order(as.numeric(as.vector(Diff$logFC))),]

diffGene=as.vector(rownames(Diff))

diffLength=length(diffGene)

afGene=c()

if(diffLength>(100)){

afGene=diffGene[c(1:50,(diffLength-50+1):diffLength)]

}else{

afGene=diffGene

}

afExp=rt[afGene,]

n=t(scale(t(afExp)))

n[n>2]=2

n[n< -2]= -2

Type=c(rep("HC",HCNum),rep("DM",DMNum))

names(Type)=colnames(rt)

Type=as.data.frame(Type)

anncolor=list(Type=c(DM=pal_npg()(1),HC=pal_npg()(2)[2]))

##pdf(file="DIFFdm_heatmap.pdf",height=7,width=8)

pheatmap(n,

annotation=Type,

color = colorRampPalette(c(pal_npg()(2)[2],"white", pal_npg()(1)))(50),

cluster_cols =F,

show_colnames = F,

scale="row",

fontsize = 8,

fontsize_row=3,

fontsize_col=8,

annotation_colors=anncolor

)

#dev.off()

P=0.05

aflogFC=0.5

Significant=ifelse((Diff$P.Value<P & abs(Diff$logFC)>aflogFC), ifelse(Diff$logFC>aflogFC,"Up","Down"), "Not")

p = ggplot(Diff, aes(logFC, -log10(P.Value)))+

geom_point(aes(col=Significant),size=3)+

scale_color_manual(values=c(pal_npg()(2)[2], "#838B8B", pal_npg()(1)))+

labs(title = " ")+

theme(plot.title = element_text(size = 16, hjust = 0.5, face = "bold"))+

geom_hline(aes(yintercept=-log10(P)), colour="gray", linetype="twodash",size=1)+

geom_vline(aes(xintercept=aflogFC), colour="gray", linetype="twodash",size=1)+

geom_vline(aes(xintercept=-aflogFC), colour="gray", linetype="twodash",size=1)

Diff$symbol=rownames(Diff)

#pdf("DIFF_vol.pdf",width=5.5,height=5)

p=p+theme_bw()

p+geom_point(size = 2, shape = 1,) +

ggrepel::geom_label_repel(

aes(label = symbol),

color="black",

label.size =0.1

)

#dev.off()

rm(list = ls())

options(stringsAsFactors = F)

getwd()

setwd("F:/r")

library(ggplot2)

library(stringr)

library(enrichplot)

library(clusterProfiler)

library(org.Hs.eg.db)

library(DOSE)

data1=read.table("DIFF_all.DM for gsea.xls",header = T,sep="\t",check.names = FALSE)

data2=read.table("DIFF_all.IS for gsea.xls",header = F,sep=",",check.names = FALSE)

GO_database <- 'org.Hs.eg.db'

gene <- bitr(data1$SYMBOL,fromType = 'SYMBOL',toType = 'ENTREZID',OrgDb = GO_database)

names(data1) <- c('SYMBOL','LogFC')

data1_merge <- merge(data1,gene,by='SYMBOL')

data1_merge<-data1_merge[,-1]

data1_merge<-na.omit(data1_merge)

data1_merge$LogFC<-sort(data1_merge$LogFC,decreasing = T)

geneList = data1_merge[,1]

names(geneList) = as.character(data1_merge[,2])

geneList

KEGG_gseresult <- gseKEGG(geneList, nPerm = 1000, minGSSize = 10, maxGSSize = 1000, pvalueCutoff=1)

ridgeplot(KEGG_gseresult,showCategory=30,label_format=90)

par(mar=c(1,1,1,1))

gseaplot2(KEGG_gseresult,1:10,pvalue_table = TRUE)

**R code 2: Stroke-related key module identification**

rm(list = ls())

options(stringsAsFactors = F)

getwd()

setwd("F:/r")

library(WGCNA)

rt<-read.table("comBatstroke.csv",sep=',',header=T)

data=rt

row.names(data)<-data$X

data<-data[,-c(1)]

data<-t(data)

powers1=c(seq(1,10,by=1),seq(12,20,by=2))

RpowerTable=pickSoftThreshold(data, powerVector=powers1)[[2]]

cex1=0.7

par(mfrow=c(1,2))

plot(RpowerTable[,1], -sign(RpowerTable[,3])*RpowerTable[,2],xlab="Soft Threshold (power)",ylab="Scale Free Topology Model Fit,signed R^2",type="n")

text(RpowerTable[,1], -sign(RpowerTable[,3])*RpowerTable[,2], labels=powers1,cex=cex1,col="red")

abline(h=0.8,col="red")

plot(RpowerTable[,1], RpowerTable[,5],xlab="Soft Threshold (power)",ylab="Mean Connectivity", type="n")

text(RpowerTable[,1], RpowerTable[,5], labels=powers1, cex=cex1,col="red")

sft <- pickSoftThreshold(data, powerVector = powers, verbose = 5)

net = blockwiseModules(

data,

power = 6,

maxBlockSize = 6000,

TOMType = "unsigned", minModuleSize = 30,

reassignThreshold = 0, mergeCutHeight = 0.25,

numericLabels = TRUE, pamRespectsDendro = FALSE,

saveTOMs = TRUE,

saveTOMFileBase = "AS-green-IS",

verbose = 3

)

table(net$colors)

mergedColors = labels2colors(net$colors)

table(mergedColors)

plotDendroAndColors(net$dendrograms[[1]], mergedColors[net$blockGenes[[1]]],

"Module colors",

dendroLabels = FALSE, hang = 0.03,

addGuide = TRUE, guideHang = 0.05)

moduleLabels = net$colors

moduleColors = labels2colors(net$colors)

MEs = net$MEs;

geneTree = net$dendrograms[[1]];

save(MEs, moduleLabels, moduleColors, geneTree,

file = "FemaleLiver-02-networkConstruction-auto.RData")

data<-t(data)

HC=44

HCData=data[,as.vector(colnames(data)[1:HC])]

IS=HC+1

ISData=data[,as.vector(colnames(data)[IS:ncol(data)])]

rt=cbind(HCData,ISData)

HCNum=ncol(HCData)

ISNum=ncol(ISData)

Type=c(rep("HC",HCNum),rep("IS",ISNum))

design <- model.matrix(~0+factor(Type))

colnames(design) <- c("HC","IS")

design = as.data.frame(design)

moduleColors <- labels2colors(net$colors)

data<-t(data)

MEs0=moduleEigengenes(data, moduleColors)$eigengenes

MEs = orderMEs(MEs0)

moduleTraitCor = cor(MEs, design , use = "p")

nSamples = nrow(data)

moduleTraitPvalue = corPvalueStudent(moduleTraitCor, nSamples)

textMatrix = paste(signif(moduleTraitCor, 2), "\n(",

signif(moduleTraitPvalue, 1), ")", sep = "")

dim(textMatrix) = dim(moduleTraitCor)

write.table(textMatrix,"textmatrix.csv",row.names=TRUE,col.names=TRUE,sep=",")

sizeGrWindow(10,8)

par(mar=c(6,13,3,3))

labeledHeatmap(Matrix = moduleTraitCor,

xLabels = colnames(design),

yLabels = names(MEs),

ySymbols = names(MEs),

colorLabels = FALSE,

colors = greenWhiteRed(50),

textMatrix = textMatrix,

setStdMargins = FALSE,

cex.text = 0.25,

cex.lab=0.5,

cex.main=1,

zlim = c(-1,1),

main = paste("Module-trait relationships"))

modNames = substring(names(MEs), 3)

geneModuleMembership = as.data.frame(cor(data, MEs, use = "p"));

write.table(geneModuleMembership,"geneModuleMembership.csv",row.names=TRUE,col.names=TRUE,sep=",")

MMPvalue = as.data.frame(corPvalueStudent(as.matrix(geneModuleMembership), nSamples));

write.table(MMPvalue,"MMPvalue.csv",row.names=TRUE,col.names=TRUE,sep=",")

names(geneModuleMembership) = paste("MM", modNames, sep="");

names(MMPvalue) = paste("p.MM", modNames, sep="")

IS=as.data.frame(design[,2]);

names(IS) = "IS"

geneTraitSignificance = as.data.frame(cor(data, IS, use = "p"));

GSPvalue = as.data.frame(corPvalueStudent(as.matrix(geneTraitSignificance), nSamples));

names(geneTraitSignificance) = paste("GS.", names(IS), sep="");

names(GSPvalue) = paste("p.GS.", names(IS), sep="")

write.table(GSPvalue,"GSPvalue.csv",row.names=TRUE,col.names=TRUE,sep=",")

module = "salmon"

column = match(module, modNames);

moduleGenes = moduleColors==module;

sizeGrWindow(7, 7);

par(mfrow = c(1,1));

verboseScatterplot(abs(geneModuleMembership[moduleGenes, column]),

abs(geneTraitSignificance[moduleGenes, 1]),

xlab = paste("Module Membership in", module, "module"),

ylab = "Gene significance for IS",

main = paste("Module membership vs. gene significance\n"),

cex.main = 1.2, cex.lab = 1.2, cex.axis = 1.2, col = module)

module = "salmon"

probes = colnames(data)

inModule = (moduleColors==module);

intModules = c("salmon")

for (module in intModules)

{

modGenes = (moduleColors==module)

modLLIDs = probes[modGenes];

fileName = paste("LocusLinkIDs-", module, ".txt", sep="");

write.table(as.data.frame(modLLIDs), file = fileName,

row.names = FALSE, col.names = FALSE)

}

lnames = load(file = "FemaleLiver-02-networkConstruction-auto.RData");

lnames

nGenes = ncol(data)

nSamples = nrow(data)

dissTOM <- 1-TOMsimilarityFromExpr(data, power = 6);

nSelect = 400

set.seed(10)

select <- sample(nGenes, size = nSelect);

selectTOM <- dissTOM[select, select];

selectTree <- hclust(as.dist(selectTOM), method = "average")

selectColors <- moduleColors[select]

sizeGrWindow(9,9)

plotDiss = selectTOM^7;

diag(plotDiss) = NA;

TOMplot(plotDiss, selectTree, selectColors, main = "Network heatmap plot, selected genes")

MEs <- moduleEigengenes(data, moduleColors)$eigengenes

IS=as.data.frame(design[,2]);

names(IS) = "IS"

MET <- orderMEs(cbind(MEs, IS))

sizeGrWindow(7,7);

par(cex = 0.9)

par(mar=c(4,4,1,1))

plotEigengeneNetworks(MET, "",

marDendro = c(0,4,1,2),

marHeatmap = c(3,4,1,2),

cex.lab = 0.8, xLabelsAngle

= 90)

rm(list = ls())

options(stringsAsFactors = F)

getwd()

setwd("F:/r")

library(ggplot2)

library(stringr)

library(enrichplot)

library(clusterProfiler)

data1=read.table("DM venn.CSV",header = T,sep=",",check.names = FALSE)

data2=read.table("IS venn.CSV",header = T,sep=",",check.names = FALSE)

GO_database<-'org.Hs.eg.db'

gene2 <- bitr(data1$DM,fromType = 'SYMBOL',toType = 'GENENAME',OrgDb = GO_database)

GO<-enrichGO( gene2$GENENAME,

OrgDb = GO_database,

keyType = "GENENAME",

ont = "ALL",

pvalueCutoff = 0.05,

qvalueCutoff = 1,

readable = T,

)

BP <- GO[GO$ONTOLOGY=='BP', ]

CC <- GO[GO$ONTOLOGY=='CC', ]

MF <- GO[GO$ONTOLOGY=='MF', ]

write.table(as.data.frame(BP), 'go.DM.BP.txt', sep = '\t', row.names = T, quote = FALSE)

write.table(as.data.frame(CC), 'go.DM.CC.txt', sep = '\t', row.names = T, quote = FALSE)

write.table(as.data.frame(MF), 'go.DM.MF.txt', sep = '\t', row.names = T, quote = FALSE)

dotplot(GO, split="ONTOLOGY",showCategory=7,font.size=8,label_format=60)+facet_grid(ONTOLOGY~., scale="free")

KEGG<-enrichKEGG(gene1$ENTREZID,

organism = "hsa",

pvalueCutoff = 0.05,

qvalueCutoff = 0.05)

save(KEGG,file = 'KEGG IS.Rdata')

barplot(KEGG,showCategory=40,font.size=8,label_format=60, title = 'KEGG enrichment analysis for IS')

**R code 3: Diabetes-related key module identification**

rm(list = ls())

options(stringsAsFactors = F)

getwd()

setwd("F:/r")

library(WGCNA)

rt<-read.table("comBatDM.csv",sep=',',header=T)

data=rt

row.names(data)<-data$X

data<-data[,-c(1)]

data<-t(data)

powers1=c(seq(1,10,by=1),seq(12,20,by=2))

RpowerTable=pickSoftThreshold(data, powerVector=powers1)[[2]]

cex1=0.7

par(mfrow=c(1,2))

plot(RpowerTable[,1], -sign(RpowerTable[,3])*RpowerTable[,2],xlab="Soft Threshold (power)",ylab="Scale Free Topology Model Fit,signed R^2",type="n")

text(RpowerTable[,1], -sign(RpowerTable[,3])*RpowerTable[,2], labels=powers1,cex=cex1,col="red")

abline(h=0.8,col="red")

plot(RpowerTable[,1], RpowerTable[,5],xlab="Soft Threshold (power)",ylab="Mean Connectivity", type="n")

text(RpowerTable[,1], RpowerTable[,5], labels=powers1, cex=cex1,col="red")

sft <- pickSoftThreshold(data, powerVector = powers, verbose = 5)

net = blockwiseModules(

data,

power = 5,

maxBlockSize = 6000,

TOMType = "unsigned", minModuleSize = 30,

reassignThreshold = 0, mergeCutHeight = 0.25,

numericLabels = TRUE, pamRespectsDendro = FALSE,

saveTOMs = TRUE,

saveTOMFileBase = "AS-green-DM",

verbose = 3

)

table(net$colors)

mergedColors = labels2colors(net$colors)

table(mergedColors)

plotDendroAndColors(net$dendrograms[[1]], mergedColors[net$blockGenes[[1]]],

"Module colors",

dendroLabels = FALSE, hang = 0.03,

addGuide = TRUE, guideHang = 0.05)

data<-t(data)

HC=63

HCData=data[,as.vector(colnames(data)[1:HC])]

DM=HC+1

DMData=data[,as.vector(colnames(data)[DM:ncol(data)])]

rt=cbind(HCData,DMData)

HCNum=ncol(HCData)

DMNum=ncol(DMData)

Type=c(rep("HC",HCNum),rep("DM",DMNum))

design <- model.matrix(~0+factor(Type))

colnames(design) <- c("HC","DM")

design = as.data.frame(design)

moduleColors <- labels2colors(net$colors)

data<-t(data)

MEs0=moduleEigengenes(data, moduleColors)$eigengenes

MEs = orderMEs(MEs0)

moduleTraitCor = cor(MEs, design , use = "p")

nSamples = nrow(data)

moduleTraitPvalue = corPvalueStudent(moduleTraitCor, nSamples)

textMatrix = paste(signif(moduleTraitCor, 2), "\n(",

signif(moduleTraitPvalue, 1), ")", sep = "")

dim(textMatrix) = dim(moduleTraitCor)

write.table(textMatrix,"textmatrixDM.csv",row.names=TRUE,col.names=TRUE,sep=",")

sizeGrWindow(14,10)

par(mar=c(6,13,3,3))

labeledHeatmap(Matrix = moduleTraitCor,

xLabels = colnames(design),

yLabels = names(MEs),

ySymbols = names(MEs),

colorLabels = FALSE,

colors = greenWhiteRed(50),

textMatrix = textMatrix,

setStdMargins = FALSE,

cex.text = 0.23,

cex.lab=0.4,

cex.main=1,

zlim = c(-1,1),

main = paste("Module-trait relationships"))

modNames = substring(names(MEs), 3)

geneModuleMembership = as.data.frame(cor(data, MEs, use = "p"));

write.table(geneModuleMembership,"geneModuleMembership.csv",row.names=TRUE,col.names=TRUE,sep=",")

MMPvalue = as.data.frame(corPvalueStudent(as.matrix(geneModuleMembership), nSamples));

write.table(MMPvalue,"MMPvalue.csv",row.names=TRUE,col.names=TRUE,sep=",")

names(geneModuleMembership) = paste("MM", modNames, sep="");

names(MMPvalue) = paste("p.MM", modNames, sep="")

DM=as.data.frame(design[,2]);

names(IS) = "DM"

geneTraitSignificance = as.data.frame(cor(data, DM, use = "p"));

GSPvalue = as.data.frame(corPvalueStudent(as.matrix(geneTraitSignificance), nSamples));

names(geneTraitSignificance) = paste("GS.", names(IS), sep="");

names(GSPvalue) = paste("p.GS.", names(DM), sep="")

write.table(GSPvalue,"GSPvalue.csv",row.names=TRUE,col.names=TRUE,sep=",")

module = "steelblue"

column = match(module, modNames);

moduleGenes = moduleColors==module;

sizeGrWindow(7, 7);

par(mfrow = c(1,1));

verboseScatterplot(abs(geneModuleMembership[moduleGenes, column]),

abs(geneTraitSignificance[moduleGenes, 1]),

xlab = paste("Module Membership in", module, "module"),

ylab = "Gene significance for DM",

main = paste("Module membership vs. gene significance\n"),

cex.main = 1.2, cex.lab = 1.2, cex.axis = 1.2, col = module)

module = "steelblue"

probes = colnames(data)

inModule = (moduleColors==module);

intModules = c("steelblue")

for (module in intModules)

{

modGenes = (moduleColors==module)

modLLIDs = probes[modGenes];

fileName = paste("LocusLinkIDs-DM", module, ".txt", sep="");

write.table(as.data.frame(modLLIDs), file = fileName,

row.names = FALSE, col.names = FALSE)

}

moduleLabels = net$colors

moduleColors = labels2colors(net$colors)

MEs = net$MEs;

geneTree = net$dendrograms[[1]];

save(MEs, moduleLabels, moduleColors, geneTree,

file = "DM-02-networkConstruction-auto.RData")

lnames = load(file = "DM-02-networkConstruction-auto.RData");

lnames

nGenes = ncol(data)

nSamples = nrow(data)

dissTOM <- 1-TOMsimilarityFromExpr(data, power = 5);

nSelect = 400

set.seed(10)

select <- sample(nGenes, size = nSelect);

selectTOM <- dissTOM[select, select];

selectTree <- hclust(as.dist(selectTOM), method = "average")

selectColors <- moduleColors[select]

sizeGrWindow(9,9)

plotDiss = selectTOM^7;

diag(plotDiss) = NA;

TOMplot(plotDiss, selectTree, selectColors, main = "Network heatmap plot, selected genes")

MEs <- moduleEigengenes(data, moduleColors)$eigengenes

DM=as.data.frame(design[,2]);

names(DM) = "DM"

MET <- orderMEs(cbind(MEs, DM))

sizeGrWindow(7,7);

par(cex = 0.9)

plotEigengeneNetworks(MET, "",

marDendro = c(0,4,1,2),

marHeatmap = c(3,4,1,2),

cex.lab = 0.8, xLabelsAngle

= 90)

rm(list = ls())

options(stringsAsFactors = F)

getwd()

setwd("F:/r")

library(ggplot2)

library(stringr)

library(enrichplot)

library(clusterProfiler)

data1=read.table("DM venn.CSV",header = T,sep=",",check.names = FALSE)

data2=read.table("IS venn.CSV",header = T,sep=",",check.names = FALSE)

GO_database<-'org.Hs.eg.db'

gene2 <- bitr(data1$DM,fromType = 'SYMBOL',toType = 'ENTREZID',OrgDb = GO_database)

GO<-enrichGO( gene2$ENTREZID,

OrgDb = GO_database,

keyType = "ENTREZID",

ont = "ALL",

pvalueCutoff = 0.05,

qvalueCutoff = 0.05,

readable = T,

)

BP <- GO[GO$ONTOLOGY=='BP', ]

CC <- GO[GO$ONTOLOGY=='CC', ]

MF <- GO[GO$ONTOLOGY=='MF', ]

write.table(as.data.frame(BP), 'go.DM.BP.txt', sep = '\t', row.names = T, quote = FALSE)

write.table(as.data.frame(CC), 'go.DM.CC.txt', sep = '\t', row.names = T, quote = FALSE)

write.table(as.data.frame(MF), 'go.DM.MF.txt', sep = '\t', row.names = T, quote = FALSE)

dotplot(GO, split="ONTOLOGY",showCategory=6,font.size=7,label_format=90,title = 'GO enrichment analysis',)+facet_grid(ONTOLOGY~., scale="free")

rm(list = ls())

KEGG<-enrichKEGG(gene2$ENTREZID,

organism = "hsa",

pvalueCutoff = 1,

qvalueCutoff = 1)

save(KEGG,file = 'KEGG DM.Rdata')

barplot(KEGG,showCategory=40, ,font.size=8,label_format=60, title = 'KEGG enrichment analysis')
